# Effect of propinox and pinaverium bromide on *ex vivo* colonic motor patterns and their synergistic effect with hyoscine butyl bromide

**DOI:** 10.3389/fphar.2025.1491123

**Published:** 2025-04-22

**Authors:** Sara Traserra, Luis Gerardo Alcalá-González, Claudia Barber, Stefania Landolfi, Carolina Malagelada, Robert Lange, Terence Appelqvist, Maura Corsetti, Marcel Jimenez

**Affiliations:** ^1^ Department of Cell Biology, Physiology and Immunology, Universitat Autònoma de Barcelona, Barcelona, Spain; ^2^ Digestive System Research Unit, Vall d’Hebron University Hospital, Barcelona, Spain; ^3^ Department of Pathology, Vall d’Hebron University Hospital, Barcelona, Spain; ^4^ Centro de Investigación Biomédica en Red de Enfermedades Hepáticas y Digestivas (CIBEREHD), Barcelona, Spain; ^5^ Opella, a Sanofi company, Frankfurt am Main, Germany; ^6^ Opella, a Sanofi company, Neuilly-sur-Seine, France; ^7^ NIHR Nottingham Biomedical Research Centre (BRC), Nottingham University Hospitals NHS Trust and the University of Nottingham, Nottingham, United Kingdom; ^8^ Nottingham Digestive Diseases Centre, University of Nottingham, Nottingham, United Kingdom

**Keywords:** abdominal pain, calcium channel blocker, colonic motility, hyoscine butylbromide, pinaverium, propinox

## Abstract

**Background:**

Antispasmodic agents are used to treat abdominal pain. The mode of action of pinaverium bromide and propinox in the colonic tissue has never been characterized. This study aimed to explore whether HBB can complement the antispasmodic effects of these drugs.

**Methods:**

Colon samples were procured from the macroscopically normal regions of 33 patients undergoing colon cancer surgery and subjected to muscle bath experiments. Pinaverium bromide and propinox alone and in combination with HBB were assessed under the following conditions: (1) spontaneous phasic contractions (SPCs) induced by isometric stretch (with 1 µM tetrodotoxin); (2) contractility induced by 10^–5^ M carbachol; (3) the electrical field stimulation (EFS) of the excitatory pathway (in the presence of 1 mM Nω-nitro-L-arginine and 10 µM MRS2179); and (4) an EFS-induced selective excitation of the inhibitory pathway (under nonadrenergic, noncholinergic pharmacological conditions). An isobolographic study was performed to evaluate the possible interaction between pinaverium bromide, or propinox and HBB.

**Results:**

Pinaverium bromide and propinox concentration-dependently reduced SPC (10^–5^ M: 29%–47% reduction) in both muscle layers. Carbachol-induced contractions were partially reduced by pinaverium bromide (10^–5^ M: 37%–46% reduction) and propinox (10^–5^ M: 32%–44% reduction) and almost totally inhibited by the combination with HBB. EFS-induced contractions were slightly decreased by pinaverium bromide (10^–5^ M; circular muscle: 39% reduction, but no effect on longitudinal muscle) and propinox (10^–5^ M: circular 48% and longitudinal 37%), and to a greater extent, by the combination with HBB. Both pinaverium bromide (10^–5^ M: 11%) and propinox (10^–5^ M: 42%) reduced the EFS-induced off-response but not the on-relaxation. The interaction index measured for the combined activity of HBB with pinaverium bromide or propinox was less than 1 in SPC, carbachol-induced contractions, and EFS-induced contractions.

**Conclusion:**

The pharmacological profile obtained in this study was consistent with an L-type calcium channel blocker for both pinaverium bromide and propinox, with an unlikely or a weak antimuscarinic effect for the latter. When combined with the antimuscarinic agent HBB, both pinaverium bromide and propinox showed a synergistic inhibition of contractile responses. This finding could have clinical implications, suggesting a combination treatment approach for greater therapeutic benefits.

## Graphical Abstract

**Figure:**
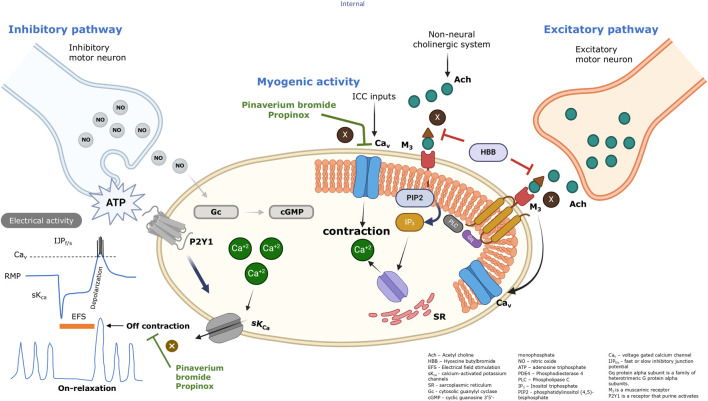
For simplicity, different postjunctional cells such as smooth muscle cells, interstitial cells and fibroblast-like cells are not shown in the diagram.

## 1 Introduction

Abdominal cramping pain is a common symptom experienced by many people. The correlation between spasmodic abdominal pain and colonic motility often stems from disturbances in the normal rhythmic contractions of the colon (Sarna, 2010). Antispasmodic medications are frequently utilized to target the relaxation of gastrointestinal (GI) smooth muscle, helping alleviate this discomfort. Drugs such as hyoscine butylbromide (HBB), pinaverium, and propinox are commonly prescribed for managing abdominal pain associated with dysfunction in the digestive and urinary tract.

Propinox, also known as pargeverine (2-(dimethylamino)ethyl diphenyl(2-propynyloxy)acetate), is an antispasmodic indicated for the treatment of spasmodic states of the digestive, hepatobiliary, and urinary tracts (Sweetman, 2007; Zheng et al., 2016). In the human gallbladder, propinox has been reported to have a dual mechanism of action, acting directly on the smooth muscle cells as a calcium channel blocker and blocking muscarinic receptors in a moderate and non-selective way (Baistrocchi et al., 1999a). However, the mode of action of propinox in the human colon has not been characterized.

Pinaverium bromide is a quaternary ammonium derivate that is also commonly used as an antispasmodic on the GI smooth muscle and indicated for the treatment of irritable bowel syndrome symptoms (Bor et al., 2021; Liu et al., 2023). Pinaverium bromide reduced contractility linked to slow wave activity in canine tissue, and it has been postulated that it acts as an L-type calcium channel blocker (Malysz et al., 1997), reducing smooth muscle contraction by preventing the calcium influx into intestinal smooth muscle cells. Patch-clamp experiments demonstrated that pinaverium reduced the voltage-dependent inward currents with an IC_50_ of 1.5 µM in the intestinal smooth muscle cells from the rabbit small intestine (Beech et al., 1990).

Hyoscine butylbromide, also referred to as scopolamine butylbromide, is a tropane alkaloid and a compound derived from the tertiary ammonium compound hyoscine. HBB is an antispasmodic drug that is globally used for relieving abdominal pain linked to muscle contractions in the GI and genitourinary systems (Corsetti et al., 2023). HBB is recognized for its action on the muscarinic receptors situated on the smooth muscle cells within the GI tract (Krueger et al., 2013; Zhang et al., 2016; Traserra et al., 2024a), leading to a relaxing and spasmolytic effect (Corsetti et al., 2023; Tytgat, 2007; Tytgat, 2008). Recent findings indicate that HBB exhibits antimuscarinic effects, inhibiting acetylcholine-induced contractions, irrespective of the origin of the mediator triggering the spasm (Traserra et al., 2024a).

Colonic tissue from macroscopically normal marginal areas, obtained from colon cancer patients, serves as a valuable biological tool for testing the effects of drugs targeting neuromuscular interactions. While variabilities related to disease, region, age, or gender may exist, *ex vivo* studies using human tissue are essential for characterizing the mechanism of action of drugs, thus eliminating the need to extrapolate from animal studies (Sanger et al., 2013). No previous studies have evaluated the modes of action of pinaverium bromide and propinox in human colonic smooth muscle tissue. Furthermore, the potential complementary effects of antispasmodics with different mechanisms of action have not been systematically explored. Our study was aimed at closing this gap by evaluating the effects of both drugs on the human colon alone and in combination with HBB.

## 2 Materials and methods

### 2.1 Human tissue collection and preparation

Human tissue samples of the right and left colon were obtained from patients undergoing colon cancer surgery at Hospital Vall d’Hebron, Barcelona, Spain, after they had provided their informed consent. The colon segments were taken from macroscopically normal marginal areas. After removal, the specimens were transported in a cold saline buffer to the laboratory at the Universitat Autònoma de Barcelona. The samples were placed in Krebs solution on a dissection dish, and the mucosal layer was carefully removed. The muscle strips (1 cm in length × 0.4 cm in width) were cut in the circular and longitudinal directions. All experimental procedures were approved by the Ethical Committee of the Universitat Autònoma de Barcelona, Barcelona, Spain (ethical approval code: 5604).

Circularly and longitudinally oriented muscle strips were studied in a 10 mL organ bath filled with carbogenated (95% O_2_ and 5% CO_2_) Krebs solution at 37°C ± 1°C. A tension of 4 g was applied, and the tissues were allowed to equilibrate for 1 h. An isometric force transducer (Harvard VF-1) connected to an amplifier was used to record the mechanical activity. Digitalized data (25 Hz) were collected using DATAWIN1 software (Panlab, Barcelona, Spain), which was coupled with an ISC-16 analog-to-digital card installed on a computer. Electrical field stimulation (EFS) was applied through two platinum electrodes placed on the support holding the tissue to study the release of inhibitory and excitatory neurotransmitters.

### 2.2 Experimental design of the study

The study was designed to evaluate the effects of pinaverium bromide and propinox; as such, four sets of experiments were conducted. HBB at a concentration of 10^–7^ M to 10^–5^ M was added to Experiments 1 to 3 to assess a possible complementary effect between drugs. Strips were randomly assigned to a protocol, with annotations for the colonic region, strip orientation (circular vs. longitudinal), gender, and age. Supplementary Table 1 displays the distribution of strips for each experiment.

#### 2.2.1 Experiment 1. Effects of pinaverium bromide and propinox on spontaneous phasic contractions

The effects of pinaverium bromide and propinox on spontaneous phasic contractions (SPCs) generated by the simple isometric stretch of the sample was studied. This type of contraction is related to ripples in an *in vivo* setting (Corsetti et al., 2019). The isolated tissue underwent a 20-min incubation with tetrodotoxin (TTX) (10^–6^ M) to inhibit neuronal activity. Pinaverium bromide and propinox were incubated at increasing concentrations ranging from 10^–9^ to 10^–5^ M. The equivalent volume of the solvent (Solutions and drugs section) used in each concentration (vehicle) served to evaluate the potential rundown of the mechanism. At the end of the experiment, after each treatment with either pinaverium bromide, propinox, or the vehicle, HBB (10^–7^, 10^–6^, and 10^–5^ M) was added to assess its impact.

#### 2.2.2 Experiment 2. Effects of pinaverium bromide and propinox on carbachol pre-contracted tissue

In this experiment, the effects of pinaverium bromide and propinox on the contraction of the circular and longitudinal smooth muscles induced by the stimulation of the muscarinic pathway with carbachol was characterized. For this purpose, the tissue was pre-incubated (20 min) with carbachol (10^–5^ M), leading to increased contractile activity. Pinaverium bromide and propinox were added to the incubations at increasing concentrations ranging from 10^–9^ to 10^–5^ M. An equivalent volume of the solvent (Solutions and drugs section) used in each concentration (as a vehicle) was employed to evaluate the potential rundown of the mechanism. At the end of each experiment with either pinaverium bromide, propinox, or the vehicle, HBB (10^–7^, 10^–6^, and 10^–5^ M) was added to assess its impact on the effect.

#### 2.2.3 Experiment 3. Effects of pinaverium bromide and propinox on neural-mediated excitatory responses

The strips of the right and left colon were subjected to incubation in non-nitrergic, non-purinergic (NNNP) conditions, comprising Nω-nitro-L-arginine (L-NNA) 10^–3^ M and MRS2179 10^−5^ M, in order to isolate excitatory neural-mediated responses. Under these specific experimental conditions, an EFS with a voltage of 30 V, a pulse duration of 0.4 ms, a frequency of 50 Hz, and a train duration of 300 ms resulted in a pronounced cholinergic sharp contraction (Traserra et al., 2024a). Pinaverium bromide and propinox were incubated at increasing concentrations ranging from 10^–9^ to 10^–5^ M. The equivalent volume of the solvent (Solutions and drugs section) used in each concentration (vehicle) was employed to evaluate the potential rundown of the mechanism. At the end of each experiment with either pinaverium bromide, propinox, or the vehicle, HBB (10^–7^, 10^–6^, and 10^–5^ M) was added to assess its impact on the effect.

#### 2.2.4 Experiment 4: Effects of pinaverium bromide and propinox on neural-mediated inhibitory responses

The strips of the right and left colon were exposed to nonadrenergic, noncholinergic (NANC) conditions, which included atropine, propranolol, and phentolamine at a concentration of 10^–6^ M to isolate inhibitory neural-mediated responses. During these experimental conditions, EFS trains (2 min) with increasing voltage (5, 6, 7, 8, 10, and 20 V) were applied at a frequency of 5 Hz and a pulse duration of 0.4 ms. This protocol was applied both before and after the incubation with pinaverium bromide and propinox at 10^–5^ M to assess the on-relaxation during the EFS and the off-contraction after the EFS. In this protocol, HBB was not tested at the end of the experiment because previous data demonstrated that HBB does not modify the EFS-induced inhibitory response or the off-response (Traserra et al., 2024a).

### 2.3 Solutions and drugs

The composition of the Krebs solution was as follows (mM): 10.10 glucose, 115.48 NaCl, 21.90 NaHCO_3_, 4.61 KCl, 1.14 NaH_2_PO_4_, 2.50 CaCl_2_, and 1.16 MgSO_4_, bubbled with a mixture of 5% CO_2_:95% O_2_ (pH 7.4). The following drugs and chemicals were used: HBB (Sanofi), pinaverium bromide (Sigma Aldrich), propinox (CymitQuimica), L-NNA (Sigma Chemicals, St. Louis, MO, United States), atropine sulfate (Sigma Chemicals, St. Louis, MO, United States), phentolamine (Sigma Chemicals, St. Louis, MO, United States), TTX (Sigma Chemicals, St. Louis, MO, United States), 2′-deoxy-N6-methyl adenosine 3′,5′-diphosphate tetraammonium salt (MRS2179) (Merck Millipore, Darmstadt, Germany), (2-hydroxyethyl)trimethylammonium chloride carbamate (carbachol) (Tocris Biosience, Bristol, United Kingdom), propranolol (Tocris, Bristol, United Kingdom), and dimethyl sulfoxide (DMSO) (Sigma Chemicals, St. Louis, MO, United States). Stock solutions were made by dissolving drugs in distilled water, except for L-NNA that required sonication to be dissolved in the Krebs solution, and pinaverium bromide and propinox which were dissolved in DMSO. DMSO was used as control (vehicle), and the maximum volume added in the organ bath was 10 μL, which means 0.1% of the total volume.

### 2.4 Data analysis

The area under the curve (AUC) (g*min) of the contractions from baseline was measured to estimate the mechanical activity before and after drug addition (Experiments 1 and 2) or before and during the inhibitory EFS (Experiment 4). In order to normalize the mechanical data, the responses to drugs and EFS-induced inhibitory on-responses were expressed as a percentage of the basal AUC using the following formula: 100 × (AUC during an EFS or after drug incubation/AUC previous to an EFS or drug addition). Thus, 0% represents the complete cessation of spontaneous motility and 100% no change compared to basal activity. For the EFS-induced excitatory responses (Experiment 3) and off-contractions (Experiment 4), the amplitude of the response was measured before and after drug addition, the data were normalized (i.e., 100% = amplitude before drug addition), and the percentage of reduction was calculated for each drug.

Since both propinox and pinaverium bromide in combination with HBB further reduced several of the measured parameters, we evaluated their possible additive or synergistic effect by means of an isobolographic study. Accordingly, we measured the interaction index based on the previously published calculation (Chou, 2006). The interaction index calculates the possible antagonistic, additive, or synergistic effect depending on whether it is less than, equal to, or greater than 1, respectively (Chou, 2006).

### 2.5 Statistical analysis

A paired two-way analysis of variance (ANOVA) was used to assess the effect of accumulative concentrations of study drugs compared with the vehicle and the effect of study drugs at 10^–5^ M on the inhibitory EFS trains of increasing voltage. Šidák’s *post hoc* test was used to assess the differences between groups. A paired one-way ANOVA was used to measure the possible complementary effect of the study drugs pinaverium bromide and propinox with HBB. Dunnett’s *post hoc* test was used to assess the differences between groups. Graph data were expressed as the mean ± standard error of the mean (SEM) and were considered significant when *p* < 0.05. In each experimental condition, the “*n*” value represented the total number of muscle strips used from different patients. For each patient, a mean of about 8 strips was studied, depending on the availability of the tissue. A statistical analysis was performed using GraphPad Prism version 6.01 (GraphPad Software, San Diego, CA, United States).

## 3 Results

Human tissue samples of the colon from macroscopically normal regions were obtained from patients (*n* = 33; 11 women and 22 men, aged 38–93 years) during colon resections for neoplasms (Table 1).

**TABLE 1:** Patient characteristics.

	Right colon	Left colon
Number of patients	17	16
Gender	4 women/13 men	7 women/9 men
Age range (mean ± SD)	38–93 years (71.9 years ± 16.67)	51–90 years (71.6 years ± 13.58)

### 3.1 Experiment 1. Effects of pinaverium bromide and propinox on SPCs

**FIGURE 1:**
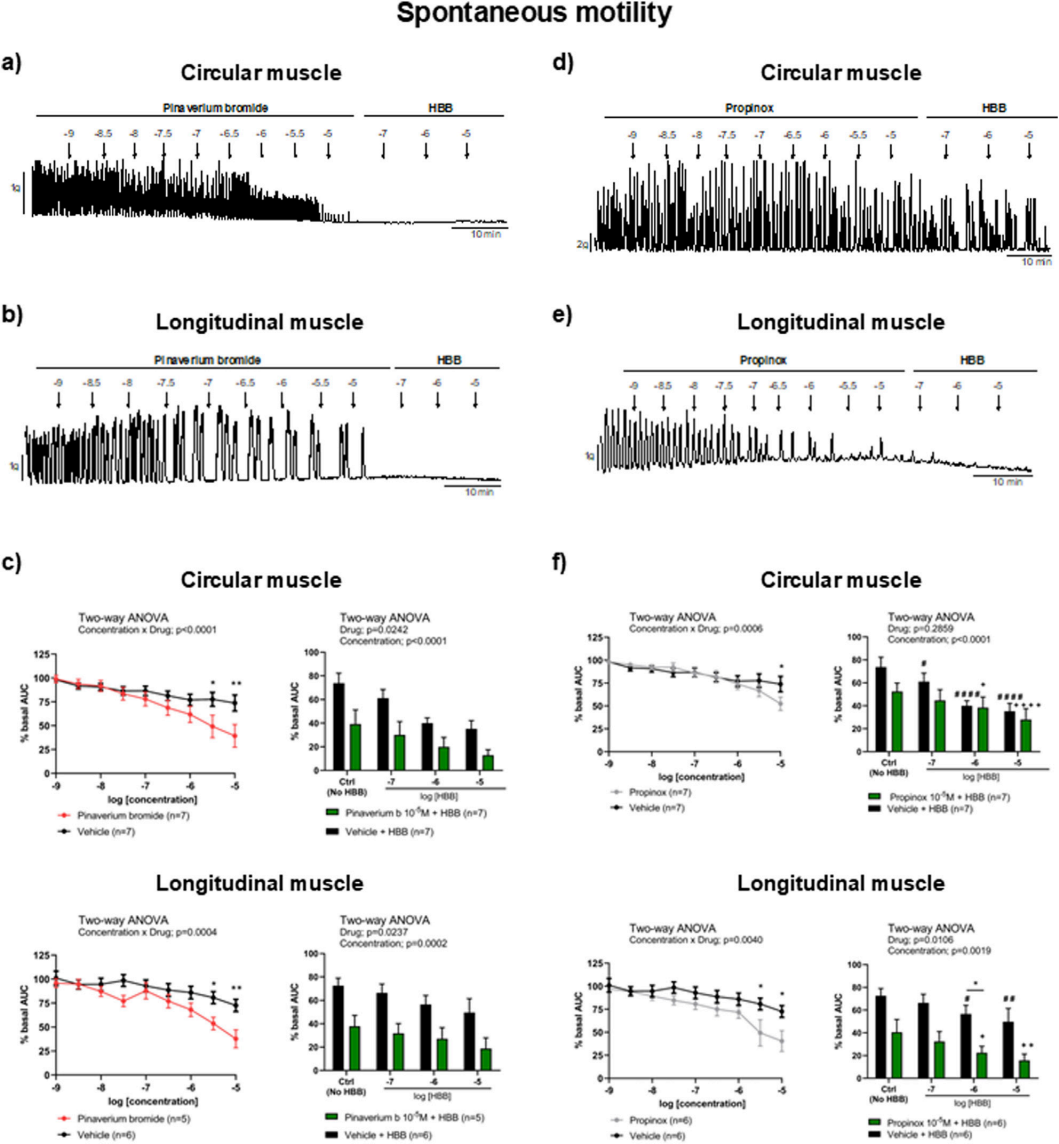
Representative mechanical recordings from the circular **(a, d)** and longitudinal **(b, e)** muscles showing the effects of pinaverium bromide **(a, b)** and propinox **(d, e)** (10^−9^–10^−5^ M) plus HBB (10^−7^–10^−5^ M) on SPCs in the presence of TTX 10^−6^ M. Graphs (left) showing the effects of accumulative concentrations of pinaverium bromide **(c)** and propinox **(f)** (10^−9^–10^−5^ M). Histograms (right) showing the effects of pinaverium bromide 10^–5^ M **(c)** and propinox 10^–5^ M **(f)** plus HBB (10^−7^–10^−5^ M) compared with HBB alone on SPCs in the circular and longitudinal muscles, respectively. Data were normalized (i.e., 100%) to the basal AUC. Data were expressed as mean ± SEM. Šidák’s *post hoc* test was done after the two-way ANOVA compared with the vehicle (*) or ctrl (^+^, ^#^). *, ^#^, ^+^ *P* < 0.05, **, ^##^, ^++^ *P* < 0.01, ^####^, ^++++^ *P* < 0.0001. ANOVA, analysis of variance; AUC, area under the curve; HBB, hyoscine butylbromide; SEM, standard error of the mean; SPC, spontaneous phasic contraction.

In the presence of the neural blocker TTX, pinaverium bromide (10^−9^–10^−5^ M) reduced SPCs compared with the vehicle. At the highest concentration, 10^–5^ M, the estimated reduction was 46.7% in the circular and 47.9% in the longitudinal muscle compared with the vehicle. The addition of HBB (10^−7^–10^−5^ M), in the presence of pinaverium bromide (10^–5^ M), resulted in a further reduction of SPCs in both muscle layers (Figures 1A, B). The reduction in SPCs was more pronounced with the combination of HBB plus pinaverium bromide (10^–5^ M) than with HBB alone in both muscle layers (Figure 1C). Similarly, propinox (10^−9^–10^−5^ M) reduced SPCs reaching a reduction of 28.9% and 44.2% (compared with the vehicle) in the circular and longitudinal muscles, respectively. The addition of HBB (10^−7^–10^−5^ M), in the presence of propinox (10^–5^ M), resulted in a further concentration-dependent reduction of SPCs. In the longitudinal muscle, the decrease in SPCs was higher with the combination of HBB plus propinox than with HBB alone (Figures 1D, E), but no such effect was observed for the circular muscle. The reduction in SPCs was more pronounced with the combination of HBB plus propinox (10^–5^ M) than with HBB alone in both muscle layers (Figure 1F). Table 2 shows the effects of the reduction on SPC obtained with pinaverium bromide alone, propinox alone, and HBB alone at 10^–5^ M compared with the vehicle.

**TABLE 2:** Estimated reductions for each drug and pattern of motility measured.

Drug	Pinaverium bromide 10^–5^ M	Propinox 10^–5^ M	HBB 10^–5^ M^a^
SPC			
Circular	46.7%^b^	28.9%	52.3%
Longitudinal	47.9%^b^	44.2%^b^	31.5%
CCH-induced contractions			
Circular	45.5%^b^	31.8%	96.4%
Longitudinal	36.9%^b^	43.7%^b^	95.9%
EFS-induced contractions			
Circular	38.8%^b^	48.2%^b^	90.8%
Longitudinal	No effect	36.9%^b^	95.0%
Off-contraction after the inhibitory response			
Circular	11%	42.5%	No effect

^a^ Data previously published in Traserra et al. (2024b), with identical experimental protocols.

^b^ In these studies, the inhibitory effect of the association of HBB, plus pinaverium bromide 10–5 M or propinox 10–5 M was higher than that of HBB, alone (Figures 1–4).

### 3.2 Experiment 2. Effects of pinaverium bromide and propinox on carbachol-induced contractions

**FIGURE 2:**
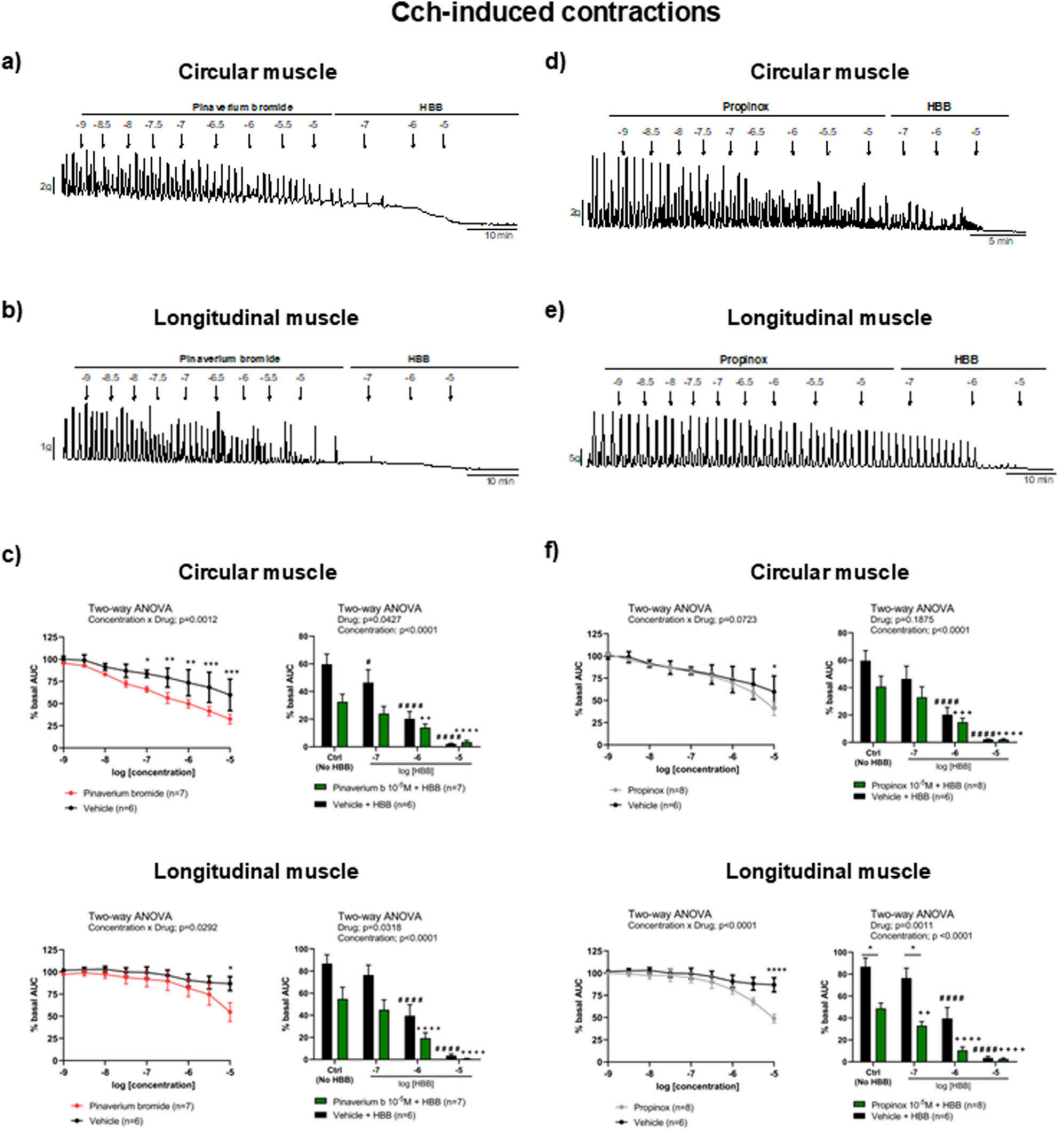
Representative mechanical recordings from the circular **(a, d)** and longitudinal **(b, e)** muscles showing the effects of pinaverium bromide **(a, b)** and propinox **(d, e)** (10^−9^–10^−5^ M) plus HBB (10^−7^–10^−5^ M) on carbachol-induced contractions. Graphs (left) showing the effects of accumulative concentrations of pinaverium bromide **(c)** and propinox **(f)** (10^−9^–10^−5^ M). Histograms (right) showing the effects of pinaverium bromide 10^–5^ M **(c)** and propinox 10^–5^ M **(f)** plus HBB (10^−7^–10^−5^ M) compared with HBB alone on carbachol-induced contractions in the circular and longitudinal muscles, respectively. Data were normalized (i.e., 100%) to the basal AUC. Data were expressed as mean ± SEM. Šidák’s *post hoc* test was done after the two-way ANOVA compared with the vehicle (*) or ctrl (^+^, ^#^). *, ^#^ *P* < 0.05, **, ^++^ *P* < 0.01, ***, ^+++^ *P* < 0.001, ****, ^####^, ^++++^ *P* < 0.0001. ANOVA, analysis of variance; AUC, area under the curve; HBB, hyoscine butylbromide; SEM, standard error of the mean.

Carbachol-induced contractions were reduced by pinaverium bromide in both muscle layers, reaching a reduction of 45.5% and 36.9% (compared with the vehicle) in the circular and longitudinal muscles, respectively. Consistent with the activation of muscarinic receptors, HBB (10^−7^–10^−5^ M) abolished carbachol-induced contractions in both muscle layers nearly completely (Figures 2A, B). The decrease in carbachol-induced contractions was more pronounced when HBB was added to pinaverium bromide at 10^–5^ M than with HBB alone in both muscle layers (Figure 2C). Similarly, propinox at 10^–5^ M reduced carbachol-induced contractions (circular: 31.8% and longitudinal: 43.7%, compared with the vehicle), and the subsequent addition of HBB abolished the contractile response nearly completely (Figures 2D, E). The combination of HBB and propinox at 10^–5^ M was more potent than HBB alone in the longitudinal muscle (Figure 2F). Table 2 summarizes the effects of the reduction on carbachol-induced contractions obtained with pinaverium bromide alone, propinox alone, and HBB alone at 10^–5^ M compared with the vehicle.

### 3.3 Experiment 3: Effects of pinaverium bromide and propinox on neural-mediated excitatory responses

**FIGURE 3:**
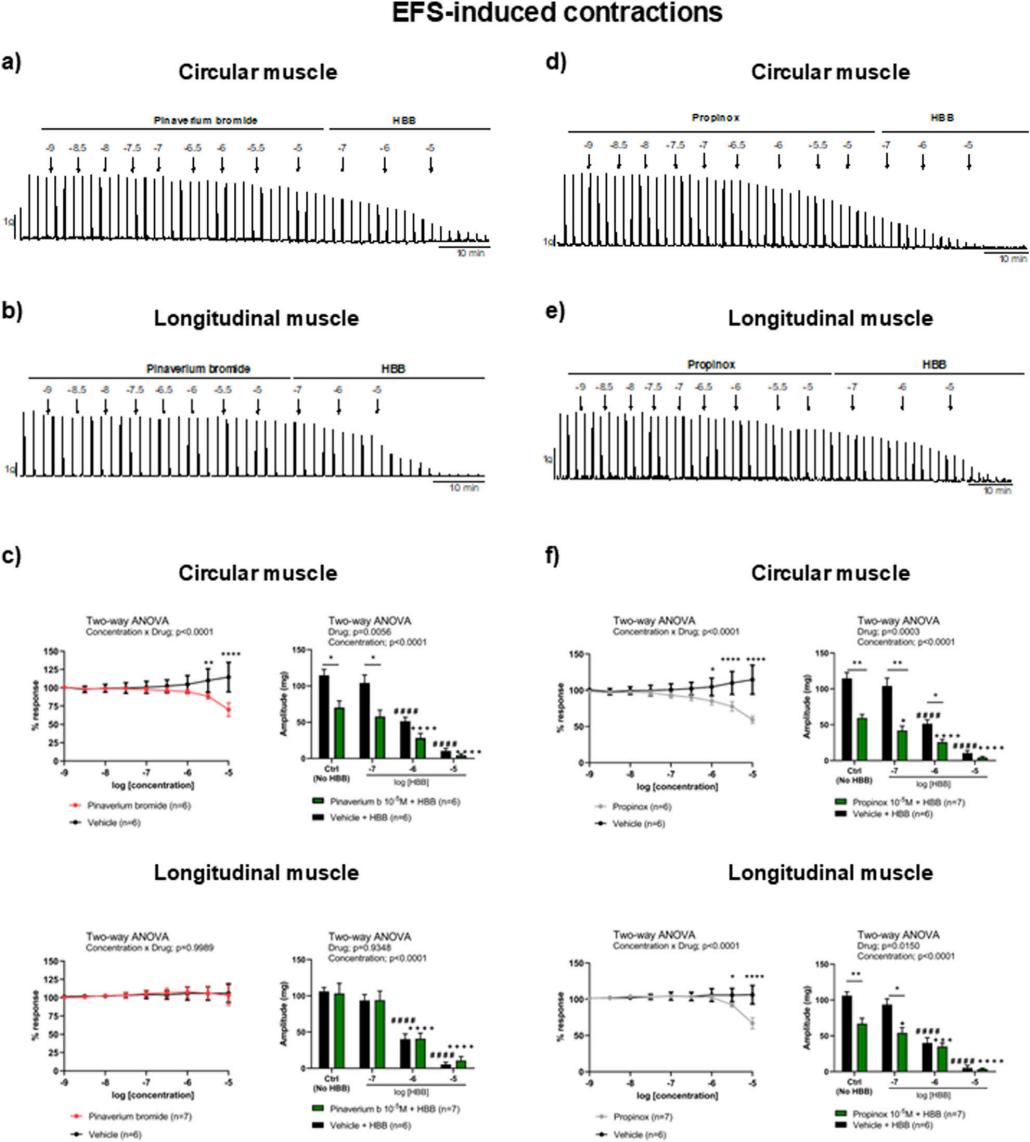
Representative mechanical recordings from the circular **(a, d)** and longitudinal **(b, e)** muscles showing the effects of pinaverium bromide **(a, b)** and propinox **(d, e)** (10^−9^–10^−5^ M) plus HBB (10^−7^–10^−5^ M) on the EFS-induced contractions in NNNC conditions. Graphs (left) showing the effects of accumulative concentrations of pinaverium bromide **(c)** and propinox **(f)** (10^−9^–10^−5^ M). Histograms (right) showing the effects of pinaverium bromide (10^−7^–10^−5^ M) **(c)** and propinox (10^−7^–10^−5^ M) **(f)** plus HBB (10^−7^–10^−5^ M) compared with HBB alone on the EFS-induced contractions in the circular and longitudinal muscles, respectively. Data were normalized (i.e., 100%) to the basal AUC. Data were expressed as mean ± SEM. Šidák’s *post hoc* test was done after the two-way ANOVA compared with the vehicle (*) or ctrl (^+^, ^#^). *, ^+^ *P* < 0.05, **P < 0.01, ***, ^+++^ *P* < 0.001, ****, ^####^, ^++++^ *P* < 0.0001. ANOVA, analysis of variance; AUC, area under the curve; EFS, electrical field stimulation; HBB, hyoscine butylbromide; SEM, standard error of the mean.

In NNNP conditions, the EFS induced a sharp contraction that was quite constant in control conditions (vehicle) during the whole experiment. Consistent with the selective activation of excitatory motor neurons, HBB alone (10^−7^–10^−5^ M) abolished the EFS responses in both muscle layers (Traserra et al., 2024a). At high concentrations, pinaverium bromide reduced the response to neural-mediated contractions only in the circular muscle (38.8%, compared with the vehicle) and not in the longitudinal muscle. When HBB was added to pinaverium bromide at 10^–5^ M, a higher reduction of neural-mediated contractions was observed in the circular muscle layer than with HBB alone (Figures 3A–C). Propinox up to 10^–5^ M reduced the response to neural-mediated contractions in both circular (48.2%) and longitudinal muscles (36.9%) compared with the vehicle. The addition of HBB to propinox at 10^–5^ M resulted in further reduction of neural-mediated contractions compared with HBB alone in both muscle layers (Figures 3D–F). Table 2 summarizes the effects of the reduction on EFS-induced contractions obtained with pinaverium bromide, propinox, and HBB at 10^−5^ M compared with the vehicle.

### 3.4 Experiment 4. Effects of pinaverium bromide and propinox on neural-mediated inhibitory responses under nonadrenergic, noncholinergic conditions

**FIGURE 4:**
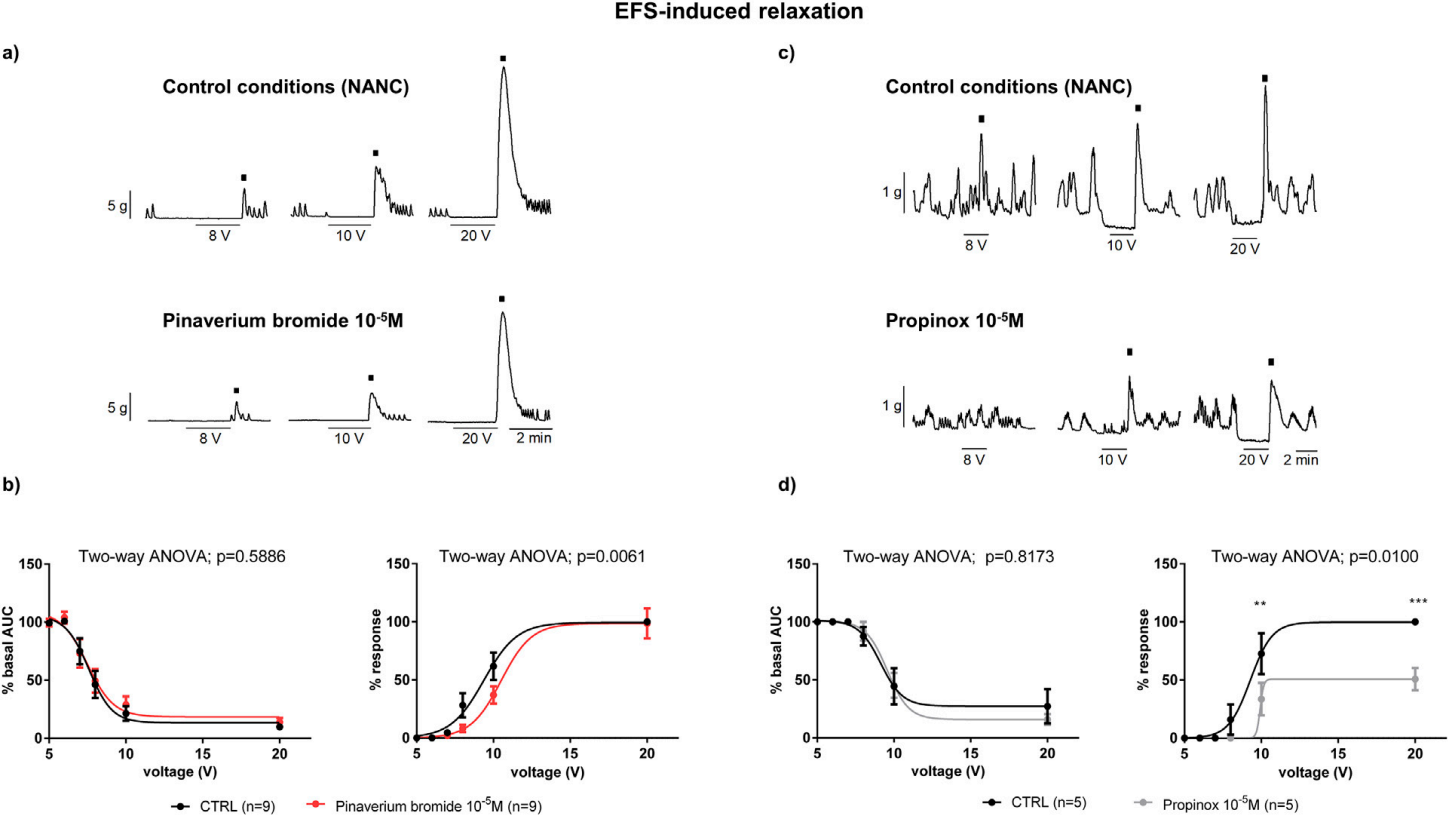
The effects of pinaverium bromide and propinox on neural-mediated inhibitory responses under NANC conditions in the circular muscle. Representative mechanical recordings showing the on-response (during the EFS) and the off-response (rebound after the EFS) in control conditions and in the presence of pinaverium bromide 10^–5^ M [**(a)** left colon] and propinox 10^–5^ M [**(c)** right colon]. Graphs showing the effects of pinaverium bromide 10^–5^ M and propinox 10^–5^ M on EFS trains of increasing voltage (5, 6, 7, 8, 10, and 20 V) on the on-response and the off-response [[**(b, d)**, respectively). On-relaxation were normalized (i.e., 100%) to the basal AUC before the EFS. Off-contractions were normalized (i.e., 100%) to the maximum response obtained with a stimulus of 20 V in control conditions. Data were expressed as mean ± SEM. Šidák’s *post hoc* test was done to compare with control. **P < 0.01, ***P < 0.001. ANOVA, analysis of variance; AUC, area under the curve; EFS, electrical field stimulation; HBB, hyoscine butylbromide; NANC, nonadrenergic, noncholinergic; SEM, standard error of the mean.

**FIGURE 5:**
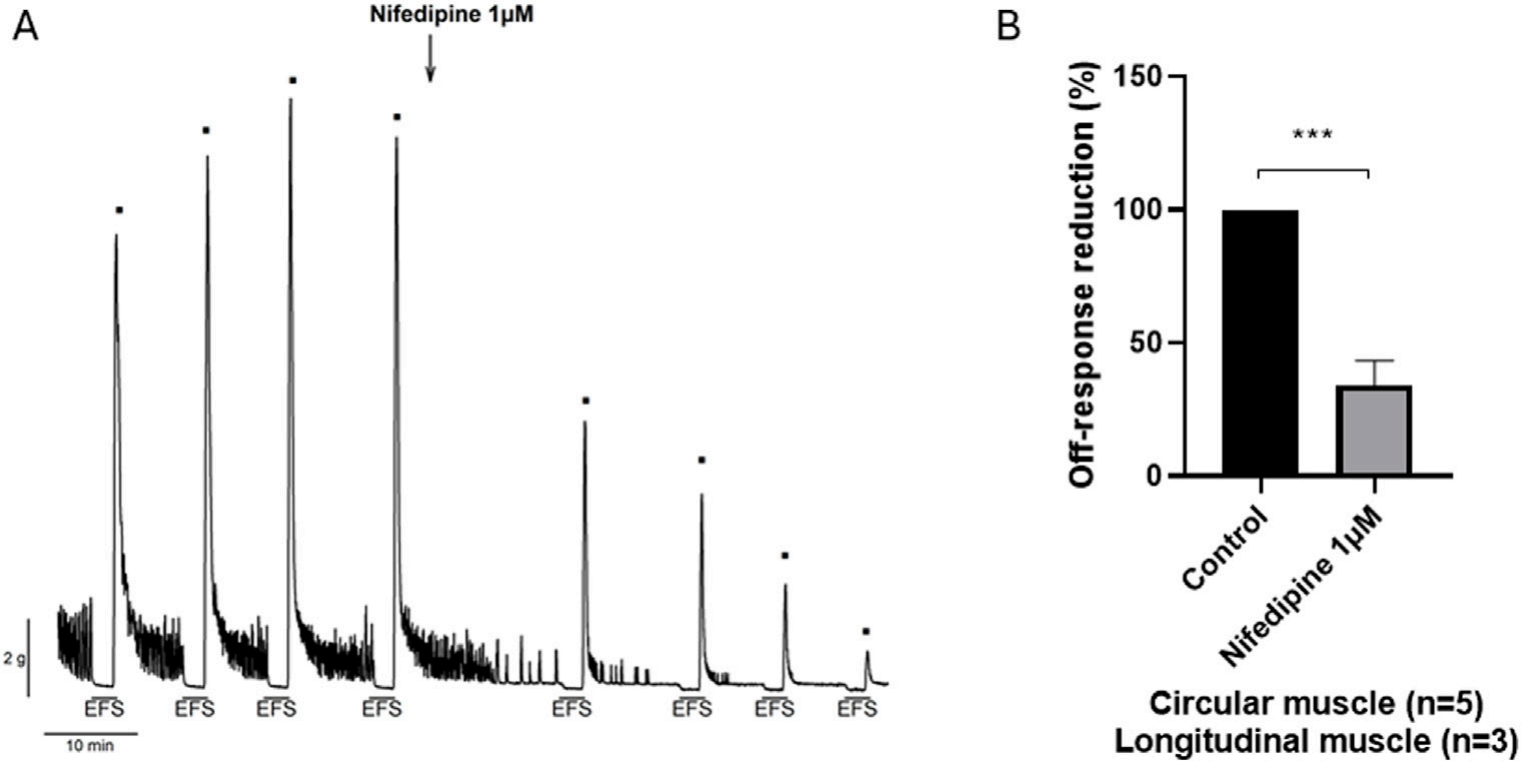
Effect of nifedipine 10^−6^ M on the off-response induced by EFS in NANC conditions **(A)**. Representative recording and **(B)**. Histogram showing the reduction in the amplitude of the off-contraction (n = 8). Data were expressed as mean ± SEM. A paired t-test was done to compare the effect before and after nifedipine addition. ***P < 0.001.

Under NANC conditions, and with a focus on the circular tissue, the EFS induced a relaxation (on-relaxation) followed by an off-contraction at the end of the stimulus. Both the on-relaxation and the off-contraction were voltage-dependent. Pinaverium bromide (10^–5^ M) slightly (11%) reduced the off-response without modifying the EFS-induced relaxation (Figures 4A, B). Propinox also reduced (42.5%) the off-contraction without any effect on the EFS-induced relaxation (Figures 4C, D). The same experiment was performed with the vehicle (*n* = 6), and no significant changes were observed, neither in the on-relaxation (*p* = 0.3634) nor in the off-contraction (*p* = 0.3804) (data not shown). Table 2 summarizes the effects of the reduction on the off-contraction measured as the reduction of the AUC obtained with pinaverium bromide and propinox at 10^−5^ M compared with the vehicle. To illustrate the strong dependence of L-type calcium channels on off-contractions, experiments were performed in which the reduction of the off-response was evaluated in the presence of 10^–6^ M Nifedipine (Figure 5).

### 3.5 Isobolographic study

**FIGURE 6:**
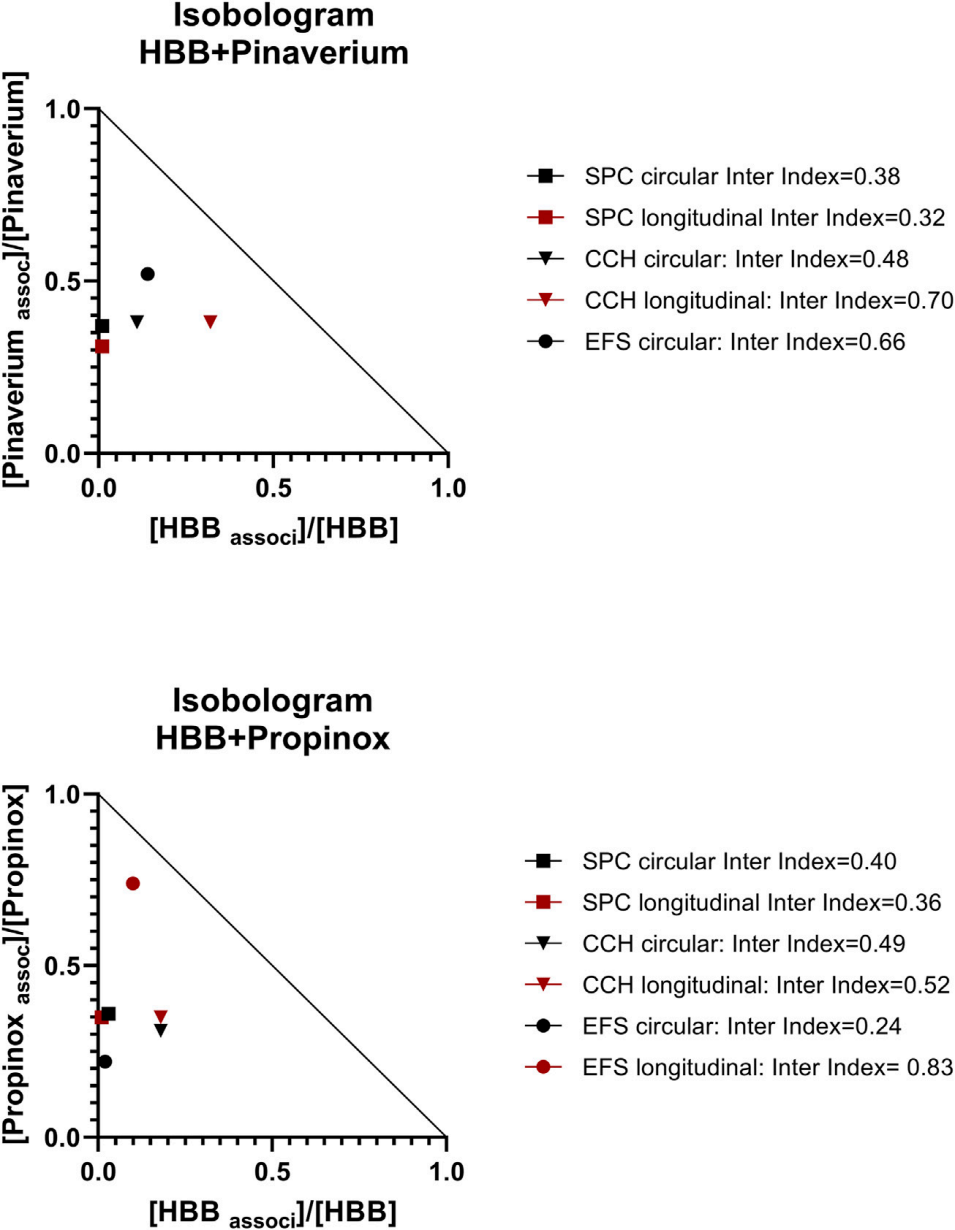
Normalized isobolograms showing the interaction index calculated with the association of HBB 10^–7^ M and pinaverium 10^–5^ M (top) or propinox 10^–5^ M (bottom). Interaction indexes were calculated for each muscle layer and for SPCs, carbachol-induced contractions, and EFS-induced contractions. Notice that the interaction index could not be calculated for the EFS-induced contractions in the longitudinal muscle as no measurable effect was detected with pinaverium alone. To build the isobologram, data for propinox or pinaverium (alone) were obtained with D-R curves (present study), and for HBB (alone), data were obtained from D-R curves from the study by Traserra et al. (2024b). Experimental conditions were identical in these two studies. Interactions index between 0.1 and 0.3 were considered strong synergism and between 0.3 and 0.7 synergism and between 0.7 and 0.85 moderate (Chou, 2006). CCH, carbachol; D-R curves, dose–response curves; EFS, electrical field stimulation; HBB, hyoscine butylbromide; SPC, spontaneous phasic contraction.

Since both pinaverium bromide and propinox (present manuscript) and HBB (Traserra et al., 2024a) reduced several of the parameters measured (Table 2), we calculated the interaction index between these two associations (Figure 6). We estimated the interaction index calculated with the mean effect of pinaverium bromide or propinox 10^–5^ M in combination with HBB 10^–7^ M. The estimated interaction index was lower than 1 in all parameters measured and in both muscle layers (Figure 6), which is consistent with a synergistic effect between HBB, propinox, and pinaverium.

## 4 Discussion

The present work is the first study that provides a comprehensive assessment of the effects of pinaverium bromide and propinox, alone and in combination with HBB, on the motility of both circular and longitudinal colonic tissue muscles *ex vivo*. Moreover, selective pharmacological conditions were used to properly study inhibitory and excitatory neuromuscular transmissions.

Our study indicated that pinaverium bromide reduced myogenic contractions and partially reduced carbachol-induced contractions in both muscle layers as well as EFS-induced contractions only in the circular muscle. Pinaverium bromide exhibited a slight reduction in the EFS-induced off-response, without altering the EFS-induced relaxation. Similar results have been obtained in tissues from animals where pinaverium-inhibited acetylcholine-triggered contractions in dog colonic smooth muscles (Malysz et al., 1997). In the rat colon, it decreased contractile responses that induced an increase in intraluminal pressure *ex vivo*. The reduction was observed after stimulation with acetylcholine or barium chloride or EFS, showing that the mode of action was probably associated with L-type calcium channel inhibition (Baumgartner et al., 1985). According to this hypothesis, the effect of pinaverium bromide has been characterized in smooth muscle cells isolated from the rabbit jejunum, where it decreased currents (IC_50_ = 1.5 µM) driven by calcium channels (Beech et al., 1990). Similar results have been reported on L-type calcium channels expressed in Chinese hamster ovary cells (Morel et al., 1998). The pharmacodynamic responses of pinaverium bromide in our study occurred within a concentration range of 10^–6^ to 10^–5^ M. These concentrations are similar to previous studies where the mode of action was assessed but higher compared to the therapeutic levels detected in plasma following the oral administration of 100 mg of pinaverium bromide (Patiño-Rodriguez et al., 2015). Similar plasma concentrations were reported after the oral administration of pinaverium bromide in Chinese healthy volunteers (Ren et al., 2011). However, it is important to note that pinaverium bromide is a quaternary ammonium derivative, and the drug can accumulate in tissues. Accordingly, the concentration found in plasma might not be the same as the concentration found in tissues (Traserra et al., 2024b) In the present study, pinaverium bromide exhibited only partial efficacy in reducing contractions induced by carbachol and the EFS. The involvement of M_3_ muscarinic receptors is pivotal in the initiation of muscle contraction, as these receptors are linked to Gq proteins, which activate phospholipase C and inositol trisphosphate, culminating in the release of calcium from intracellular stores. The stimulation of M_3_ receptors on the muscle cell membrane results in smooth muscle depolarization and the opening of L-type calcium channels. Consequently, pinaverium bromide specifically attenuates the contraction associated with the activation of voltage-gated calcium channels but is unlikely to influence the pathway linked to the release of calcium from intracellular stores. As a result, it only partially mitigates the contractile response. Accordingly, the effect of HBB was examined alone (Traserra et al., 2024a) and in combination with pinaverium bromide. In these conditions, HBB alone strongly (90%–95%) reduced the responses induced by carbachol and the EFS, confirming the involvement of muscarinic receptors in this response (Traserra et al., 2024a). The association of HBB and pinaverium bromide was more effective in reducing contractions than HBB alone in both muscle layers in carbachol-induced contractions, whereas a synergistic effect was observed just in the circular muscle after the EFS-induced contractions. The differences between the EFS-induced contractions and carbachol responses might be due to the activation of junctional receptors when neurons are stimulated after an EFS and both junctional and extra junctional receptors are stimulated with carbachol (Drumm et al., 2020). Moreover, it is important to note that pinaverium bromide up to 10^–5^ M was ineffective in the reduction of EFS responses in the longitudinal muscle, whereas at the same concentration, HBB abolished the EFS-induced responses, suggesting a minor role of L-type calcium channels in the contractile responses in this muscle layer.

In our study, the off-contraction, which is unrelated to muscarinic receptors, was observed in the presence of 10^–6^ M atropine in an *ex vivo* setting, indicating its independence from cholinergic inhibition. Despite atropine, the EFS induced an inhibitory response followed by an off-contraction, demonstrating that the off-contraction persists under NANC conditions. Previous research from our group delineated the co-transmission involving nitric oxide (NO) and a purine acting on P2Y_1_ receptors (Gallego et al., 2006; Gallego et al., 2011), where NO activates cytosolic guanylyl cyclase, leading to increased cyclic guanosine 3′5′-monophosphate. Purines activate P2Y_1_ receptors, releasing calcium and activating small-conductance calcium-activated potassium channels. P2Y_1_-receptor activation contributes to the fast inhibitory junction potential (IJP), while NO induces the slow IJPs (Gallego et al., 2008), collectively forming the electrophysiological basis for smooth muscle relaxation (Mañé et al., 2014). Following stimulus cessation, smooth muscle repolarization and the opening of L-type calcium channels trigger an off-contraction. Consistent with this hypothesis, pinaverium bromide and nifedipine both partially reduced the off-contraction.

It has been previously reported that otilonium bromide, another quaternary ammonium derivate, inhibits the current associated with L-type calcium channels in isolated cells (Martin et al., 2004) and decreases the rise of intracellular calcium after several stimuli (Martínez-Cutillas et al., 2013). Also, in the human sigmoid colon, otilonium bromide partially decreased on- and off-contractions, although pan-neural stimulation was performed, and therefore, it was difficult to distinguish between both responses (Gallego et al., 2010). In the present study, through the selective stimulation of inhibitory and excitatory motor neurons, we were able to define the impact of pinaverium bromide more precisely in comparison with previous studies.

It has been reported that the administration of pinaverium bromide alone or in combination with an intramuscular injection of HBB 1 day before a nonanesthetic colonoscopy was beneficial in relieving symptoms of abdominal pain during the exploration (Wang et al., 2017). This may align with the synergistic mechanism of action of pinaverium bromide with HBB, consistent with a spasmolytic effect due to inhibition of L-type calcium channels and muscarinic receptors, respectively.

Propinox reduced myogenic contractions and partially reduced carbachol- and EFS-induced contractions in both muscle layers. It also reduced the off-contraction, although no effect was observed on the EFS-induced relaxation. As mentioned previously, the off-contraction is independent of muscarinic receptors (Traserra et al., 2024a), and therefore, the mode of action of propinox is possibly attributable to L-type calcium channel inhibition. Previous studies suggested that propinox might have a dual mode of action, binding with muscarinic receptors and calcium channels. A previous study showed that propinox, with an EC_50_ of 1.25 × 10^−7^ M, reduced carbachol (10^–4^ M)-induced contractions in an isolated human gall bladder and showed affinity for muscarinic receptors (M1, M2, and M3) as well as L-type calcium channels (Baistrocchi et al., 1999a). The potency was compared with other antispasmodics, obtaining the following rank of order inhibiting carbachol-induced contractions: atropine (EC_50_: 5.03 × 10^−8^ M) > propinox (1.25 × 10^−7^ M) > verapamil (6.63 × 10^−6^ M) > HBB (5.4 × 10^−5^ M). The effect of propinox has also been assessed in the Guinea pig gallbladder. In this case, less inhibition of carbachol-induced contractions was observed, with a relative potency that was 20.22-fold less than that in the human model (Baistrocchi et al., 1999b).

In the present study, we could not reproduce these results in the human colon. Propinox only partially reduced carbachol- (at 10^–5^ M) and EFS-induced contractions (10^−6^–10^−5^ M), whereas the addition of HBB (10^−7^–10^−5^ M) strongly reduced both responses. Compared with the results previously obtained with atropine and HBB in the human colon (Traserra et al., 2024a), the rank of potency in this tissue is atropine > HBB > propinox, both in the EFS- and carbachol (10^–5^ M)-induced contractions.

According to the aforementioned results, the effects of propinox might be attributable to L-type calcium channel inhibition causing the suppression of myogenic activity and the off-contractions associated with smooth muscle relaxation. The reduction observed in carbachol-induced contractions and EFS-induced responses might also be attributable to L-type calcium inhibition, with a weak effect on muscarinic receptors. The effect obtained with the association of HBB and propinox was more potent than that of HBB alone in some experiments, suggesting that the spasmolytic effects of HBB and propinox are probably synergistic inhibition of muscarinic receptors and L-type calcium channels, respectively.

For the time being, it is not possible to correlate the concentrations used in this study with plasma concentrations *in vivo* as there is no published information available on plasma levels of propinox.

It is important to note that the mechanism of action of these drugs can be linked to their effects on various cell types. Smooth muscles are responsible for generating the force of contraction, making them the final mediators of spasm. However, muscle contractions are strongly regulated by interstitial cells, such as ICCs and PDGFRα+ cells. Smooth muscle cells are electrically coupled to ICCs and PDGFRα+ cells, forming a SIP (Smooth muscle, PDGFRα+ cells, and ICC) syncytium (Koh et al., 2012). It is well established that ICCs serve as pacemaker cells in the gut and, along with PDGFRα+ cells, participate in neuromuscular transmission. For instance, excitatory cholinergic neuromuscular transmission has been linked to the activation of M3 receptors in intramural ICCs (Drumm et al., 2020). In this context, the spasmolytic effect of HBB may be related to the inhibition of this mechanism. Spasmolytic drugs targeting L-type calcium channels not only reduce the development of muscle contractions but may also impact slow waves originating in ICCs. Indeed, the expression of genes encoding L-type calcium channels (Cacna1c) has been observed in various subclasses of ICCs. Due to the interaction of various cell types in the development of spontaneous phasic contractions, the term “myogenic” has been questioned, and the term “SIPgenic” has been proposed instead (Sanders et al., 2024). In this manuscript, a detailed analysis of the Ca^2+^ dynamics within the SIP syncytium is provided, showing that spasmolytic agents that reduce myogenic activity may act on different cell types (Sanders et al., 2024). Other potential drug effects on the SIP syncytium, such as intracellular calcium release in various cell types or impacts on neural pathways, require further investigation.

## 5 Conclusion

Both pinaverium bromide and propinox reduced spontaneous myogenic contractions and partially reduced carbachol- and EFS-induced contractions in the colonic tissue. Both also slightly reduced the off-response without any impact on the EFS-induced relaxation. HBB partially decreased spontaneous myogenic contractions, and in contrast, HBB strongly reduced carbachol- and EFS-induced responses. Propinox has a pharmacological profile like pinaverium, which is consistent with an L-type calcium channel blocker and may have a synergistic mechanism of action with HBB (inhibiting muscarinic receptors) to provide smooth muscle relaxation. However, the possible antimuscarinic effect previously described is unlikely or very weak. This could carry significant clinical implications as a combined treatment approach may provide therapeutic advantages not observed with a singular therapy.

## Data Availability

The raw data supporting the conclusions of this article will be made available by the authors, without undue reservation.
