# Supplementary material for: Effect of propinox and pinaverium bromide on ex vivo colonic motor patterns and their synergistic effect with hyoscine butyl bromide
Source: Front Pharmacol. 2025 Apr 22;16:1491123. doi: 10.3389/fphar.2025.1491123 (PMC12052706; doi:10.3389/fphar.2025.1491123)
Supplement: Supplementary file 1 [file Table1.docx]

Suplementary table 1. Colonic Strips studied with Pinaverium Bromide and Propinox in each protocol.

| Pinaverium Bromide | | | | |
| --- | --- | --- | --- | --- |
| Protocol | n | Right/left Colon | F/M | Age |
| Experiment 1 |  |  |  |  |
| circular | 7 | 5/2 | 1/6 | 69.9±6.8 |
| longitudinal | 5 | 2/3 | 4/1 | 57.4±6.5 |
| Experiment 2 |  |  |  |  |
| Circular | 7 | 4/3 | 0/7 | 61.7±6.4 |
| longitudinal | 7 | 5/2 | 0/7 | 63.0±7.5 |
| Experiment 3 |  |  |  |  |
| circular | 6 | 4/2 | 1/6 | 59.0±7.5 |
| longitudinal | 7 | 2/5 | 1/6 | 65.6±4.7 |
| Experiment 4 |  |  |  |  |
| circular | 9 | 2/6 | 5/4 | 71.0±5.8 |

| Propinox | | | | |
| --- | --- | --- | --- | --- |
| Protocol | n | Right/left Colon | F/M | Age |
| Experiment 1 |  |  |  |  |
| circular | 7 | 3/4 | 3/4 | 72.9±7.0 |
| longitudinal | 6 | 4/2 | 2/4 | 71.7±3.8 |
| Experiment 2 |  |  |  |  |
| Circular | 8 | 4/4 | 1/7 | 67.0±3.6 |
| longitudinal | 8 | 3/5 | 5/3 | 76.3±6.7 |
| Experiment 3 |  |  |  |  |
| circular | 6 | 4/2 | 2/4 | 69.0±7.7 |
| longitudinal | 7 | 2/5 | 5/2 | 80.9±5 |
| Experiment 4 |  |  |  |  |
| circular | 5 | 2/3 | 3/2 | 72.6±5.2 |

In each protocol, the number of strips (n), the Colonic Region (Right/left), gender (F/M), and age (y±sem) were studied with Pinaverium Bromide (Top) and Propinox. (Bottom). A similar Distribution was performed with the vehicle of each drug (not shown). Notice that strips were randomly assigned to a protocol.
